# Brazilian science: towards extinction?

**DOI:** 10.1590/0074-02760210357

**Published:** 2022-02-09

**Authors:** Samuel Goldenberg, Marcio L Rodrigues, Wilson Savino

**Affiliations:** 1 Fundação Oswaldo Cruz-Fiocruz, Instituto Carlos Chagas, Curitiba, PR, Brasil; 2 Universidade Federal do Rio de Janeiro, Instituto de Microbiologia Paulo de Góes, Rio de Janeiro, RJ, Brasil; 3 Fundação Oswaldo Cruz-Fiocruz, Instituto Oswaldo Cruz, Rio de Janeiro, RJ, Brasil

**Keywords:** Brazilian science, funding policies, federal budget

## Abstract

Brazilian science is under attack. In this manuscript, we will discuss the most recent events that, if not reverted, will make Brazilian science inviable. We urge the scientific community in Brazil and abroad to stand up and resist in defense of more than a century of essential scientific contributions.

October 8th, 2021, will be remembered in Brazil by two major catastrophic events (which, as seen below, are interconnected): official numbers reported more than six-hundred thousand Coronavirus disease 19 (Covid-19) deaths. On the very same day, the Brazilian parliament approved a proposal of the Federal government reducing in 92% an already insufficient budget for the Ministry of Science & Technology. Unfortunately, such catastrophes were like a “chronicle of a death foretold” and are ultimately associated because both are related to the low priority of science and scientific knowledge by the current policies of the Brazilian government.

Funding Brazilian science is essentially done by governmental resources, handled by the federal agencies such as the National Council for Scientific and Technological Development (Conselho Nacional de Desenvolvimento Científico e Tecnológico - CNPq - www.cnpq.br), Coordination for the Improvement of Higher Education Personnel (Coordenação de Apoio ao Ensino Superior - CAPES - www.capes.gov.br, aiming to support Universities and graduate activities), and Financiadora de Estudos e Projetos (FINEP) (www*.*finep.gov.br, created to induce initiatives related to the interactions of research with the industrial sector). CNPq and CAPES are 70 years old whereas FINEP was created in 1967, indicating that science became a governmental concern quite recently in Brazil, with the creation of the Brazilian Ministry of Science and Technology (www.mcti.gov.br) in March 1985. Exceptionally, research in a few Brazilian states is efficiently supported by local foundations.

Most of the scientific activities in Brazil are developed in public Universities or Research Institutes and during the 2000 decade, the budget to support science in these institutions ranged between 1.0 to 1.3% of the gross national product (GNP). However, since 2018 there was an important decrease in the budget for research and development (R&D) activities and the investment in 2020 was lower than that of the year 2000, considering the budget in U$ dollars (Figure). Although two decades outdated, and despite the growth of the science, technology and education system, the budget was still submitted to the 92% reduction announced on October 8th. This scenario contrasts with the expansion of the Brazilian science reported by Nature Medicine 10 years ago,^1^ and with the protagonist role played by Brazilian scientists in recent health emergencies, including the Zika virus epidemic.^2^

**Figure:**
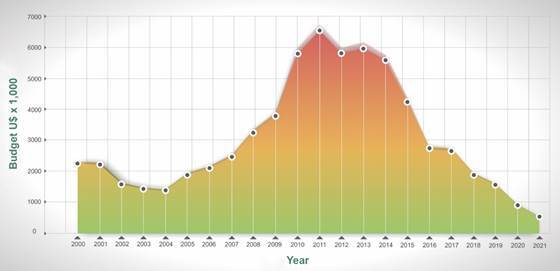
Federal budget for science and technology in Brazil in the last 20 years (U$ dollars x 1,000). The lowest numbers were reached in 2021, which did not impede the Federal Government to impose a 92% cut for 2022. This figure was kindly prepared by Wagner Nagib (Carlos Chagas Institute - Fiocruz).

Along with this shortage in the direct income for science, the budget for federal universities ― the most well ranked and, together with state universities, responsible for 95% of the scientific activity in Brazil^3^ ― was strongly affected, resulting in the scrapping of the laboratories, lack of personnel and interruption of research activities. It is estimated that approximately 80% of the scientific knowledge in Brazil is produced in the context of Graduate Programs.^4^ Still, there was a reduction in the offer of fellowships to graduate students, whose nominal values are low and have not been changed since 2015, despite the inflation that reached 10% for the last 12 months.

A short-term consequence of this dismantling policy in the Brazilian science is that the brain drain is enormously increasing among young researchers, jeopardising all previous efforts and investments in their professional formation.^5^ ^,^ ^6^ This is a clear demonstration of waste of federal resources, and a direct consequence is the enormous frustration in those building their way in science, leading them to interrupt their carriers. The long-term consequence of this attack to science is that knowledge is a commodity nowadays, and it is being burned to a similar extent that forests are burned in Brazil. Also, we foresee a loss in the criticism in the educational process of youth, thus negatively impacting future generations.

In respect to the Covid-19 pandemic, scientific evidence was not applied for establishing federal public policies to coordinate the national efforts to control the spread of the virus. Such a denialist position surely contributed to the absurd number of lives already taken in Brazil. It is estimated that at least two thirds out of these more than 600,000 Covid-related deaths in Brazil could be avoided by early vaccination, in addition to the adoption by the Ministry of Health of the well-established sanitary procedures used for the control of respiratory diseases.^7^

Having described the present conditions, should we state that the extinction process of science and technology in Brazil is already irreversible? Not yet, we think, and we hope. The scientific community in Brazil has proven to be strong enough to protect and expand the legacy of Oswaldo Cruz, Carlos Chagas, Cesar Lattes and others. Nevertheless, it urges a national and international movement to stop the ongoing process NOW. Once stopped, Brazilian researchers, together with the scientific community worldwide, must be united so that to rebuild the inflorescence of science in our country, together with education and critical thinking. This will certainly take time, but we, citizens of Brazil, have no other choice.

## References

[1] (2011). Biomedicine in Brazil. Nat Med.

[2] Barreto ML, Barral-Netto M, Stabeli R, Almeida-Filho N, Vasconcelos PFC, Teixeira M (2016). Zika virus and microcephaly in Brazil a scientific agenda. Lancet.

[3] UNIFESP (2019). Universidades públicas realizam mais de 95% da ciência no Brasil. https://www.unifesp.br/noticias-anteriores/item/3799-universidades-publicas-realizam-mais-de-95-da-ciencia-no-brasil.

[4] SBPC (2018). 80% da pesquisa no Brasil está ligada a programas de pós-graduação. http://portal.sbpcnet.org.br/noticias/80-da-pesquisa-no-brasil-esta-ligada-a-programas-de-pos-graduacao-2/.

[5] (2021). The Economist. Brazil's brain drain is getting worse. Political instability and a shortage of funds are pushing scientists abroad. https://www.economist.com/the-americas/2021/07/24/brazils-brain-drain-is-getting-worse.

[6] Leal J (2020). Politics, COVID and Brain Drain in Bolsonaro's Brazil. https://www.if.org.uk/2020/07/06/politics-covid-brain-drain-in-brazil/.

[7] Christian H (2021). CPI: 400 mil mortes poderiam ter sido evitadas, dizem especialistas. Senado Federal.

